# Integrating HIV testing into cervical cancer screening in Tanzania: an analysis of routine service delivery statistics

**DOI:** 10.1186/1472-6874-14-120

**Published:** 2014-09-30

**Authors:** Marya Plotkin, Giulia VR Besana, Safina Yuma, Young Mi Kim, Yusuph Kulindwa, Fatma Kabole, Enriquito Lu, Mary Rose Giattas

**Affiliations:** Jhpiego, New Bagamoyo Road, P.O. Box 9170, Dar es Salaam, Tanzania; Ministry of Health and Social Welfare, Reproductive Health Cancer Unit, Dar es Salaam, Tanzania; Jhpiego, Baltimore, MD USA

**Keywords:** Cervical cancer prevention, Cervical cancer screening, HIV counseling and testing, Integrated health services, Tanzania

## Abstract

**Background:**

While the lifetime risk of developing cervical cancer (CaCx) and acquiring HIV is high for women in Tanzania, most women have not tested for HIV in the past year and most have never been screened for CaCx. Good management of both diseases, which have a synergistic relationship, requires integrated screening, prevention, and treatment services. The aim of this analysis is to assess the acceptability, feasibility and effectiveness of integrating HIV testing into CaCx prevention services in Tanzania, so as to inform scale-up strategies.

**Methods:**

We analysed 2010 – 2013 service delivery data from 21 government health facilities in four regions of the country, to examine integration of HIV testing within newly introduced CaCx screening and treatment services, located in the reproductive and child health (RCH) section of the facility. Analysis included the proportion of clients offered and accepting the HIV test, reasons why testing was not offered or was declined, and HIV status of CaCx screening clients.

**Results:**

A total of 24,966 women were screened for CaCx; of these, approximately one-quarter (26%) were referred in from HIV care and treatment clinics. Among the women of unknown HIV status (n = 18,539), 60% were offered an HIV test. The proportion of women offered an HIV test varied over time, but showed a trend of decline as the program expanded. Unavailability of HIV test kits at the facility was the most common reason for a CaCx screening client not to be offered an HIV test (71% of 6,321 cases). Almost all women offered (94%) accepted testing, and 5% of those tested (582 women) learned for the first time that they were HIV-positive.

**Conclusion:**

Integrating HIV testing into CaCx screening services was highly acceptable to clients and was an effective means of reaching HIV-positive women who did not know their status; effectiveness was limited, however, by shortages of HIV test kits at facilities. Integration of HIV testing into CaCx screening services should be prioritized in HIV-endemic settings, but more work is needed to eliminate logistical barriers. The coverage of CaCx screening among HIV care and treatment-enrolled women in Tanzania may be low and should be examined.

## Background

Tanzanian women face a dual burden of disease: rates of both cervical cancer and HIV/AIDS are high. Cervical cancer (CaCx) is the most common and deadly cancer among women; each year, over 7,300 Tanzanian women are diagnosed with the disease and more than half die [1]. The age-adjusted incidence rate of cervical cancer in Tanzania is among the highest in the world at 54 deaths per 100,000 women, compared with 42.7 across Eastern Africa and 14 worldwide [1]. HIV/AIDS is also among the leading causes of death for women in Tanzania, with HIV prevalence estimated at 6.2% among women age 15–49 [2].

Screening programs for cervical cancer and HIV can save lives when linked with appropriate treatment. Inspecting the cervix after applying a dilute solution of acetic acid allows for the visualization, identification, and immediate treatment of precancerous lesions with cryotherapy, which has an 86% to 95% cure rate for eligible lesions [3]. In developing countries, a one-time screening at around age 35 has been shown to reduce a women’s lifetime risk of cervical cancer by 25% to 36% [4]. Similarly, providing anti-retroviral therapy to people infected with HIV has been shown to increase life expectancy by 11 years in South Africa, while reducing HIV transmission [5]. Treating HIV with anti-retroviral therapy also reduces mortality due to cervical cancer, according to modelling based on a cohort in Cameroon [6].

To achieve a significant public health impact, cervical cancer and HIV screening must be implemented on a wide scale [7–9], but this has proven difficult to achieve in many developing countries. While estimates vary [10, 11], a 2008 analysis of population-based surveys in 57 countries found that 19% of women in developing countries, on average, had been screened for cervical cancer in the preceding three years. Although Tanzania was not included in this analysis, neighbouring countries such as Malawi, Kenya and Zambia had among the lowest screening rates, with more than 90% of women reporting that they had never had a pelvic exam [12]. HIV testing also remains far from universal among Tanzanian women. While the proportion of women who reported *ever* being tested for HIV increased from 37% in 2007 [13] to 62% in 2011 [2], only 30% of women surveyed in 2011 reported that they had been tested in the last 12 months and had received the test results.

Integrating health services is a proven strategy for improving quality of care and increasing uptake of services. It is particularly relevant to cervical cancer and HIV/AIDS, because both are sexually transmitted and infection with one increases vulnerability to the other [14]. HIV-positive women are at greater risk of infection with human papilloma virus (HPV), the main cause of invasive cervical cancer [15, 16], and HIV infection is associated with greater prevalence and persistence of HPV, high-risk HPV subtypes, and higher HPV viral load [17, 18]. Likewise, women with HPV are more vulnerable to HIV infection, as seen in a study in Zimbabwe which noted a 2.4 times greater risk of acquiring HIV infection among women with cervical HPV infection [19].

Dozens of countries have adopted integrated approaches to expand HIV testing coverage, instructing providers to routinely offer HIV tests to patients seeking antenatal, family planning, outpatient, or other services [20–24]. The Tanzanian Ministry of Health and Social Welfare (MOHSW) integrated this type of provider-initiated HIV testing and counselling (PITC) into routine health service delivery in 2007 [25]. While numerous implementation challenges have been noted, PITC has succeeded in increasing the number of people tested for HIV and identifying previously undiagnosed cases [22, 26, 27]. The success of PITC in antenatal care—including in Tanzania—suggests that integrating PITC into cervical cancer screening could be a successful approach to increasing uptake of HIV testing among women, with the goal of initiating HIV care and treatment at an earlier stage. Documented results, however, are scarce. The cervical cancer prevention program in Zambia, for example, has adopted PITC, but has not yet published results in peer-reviewed literature [20].

Until recently in Tanzania, testing for cervical cancer was limited to just two tertiary-level facilities and was only available to women with suspected cancer. In 2010 the MOHSW announced a National Strategy for Cervical Cancer Prevention [28] that recommends a low-cost approach proven to be highly effective in developing countries: visual inspection with acetic acid (VIA) to screen women, followed by immediate cryotherapy or referral for loop electrosurgical excision procedure (LEEP) when pre-cancerous lesions are found [29–32]. In the National Strategy, the MOHSW recommends that VIA screening and cryotherapy be offered primarily at Reproductive and Child Health (RCH) clinics and that PITC be integrated into cervical cancer screening. The recommendation of the MOHSW was based on a priority to offer cervical cancer screening to all women, regardless of HIV status. In contrast, most other Sub-Saharan African countries that have integrated cervical cancer and HIV services have located VIA screening in HIV care and treatment clinics and prioritized HIV-positive women for screening [30, 33–35].

To assess the acceptability of the integration of HIV testing into cervical cancer screening and treatment services, we conducted a secondary analysis on routine service delivery data from selected government health facilities in Tanzania implementing CaCx screening and treatment services. These facilities offered CaCx screening using VIA, cryotherapy for eligible clients, referral for women with large lesions or suspect cancer, and offered HIV testing as part of the service. Three of the facilities (regional and national referral hospitals) additionally offered LEEP for treatment of large lesions.

The objective of this analysis was to provide a rough measure of the success of integration of HIV testing into cervical cancer screening, in order to inform scale-up of cervical cancer screening services in Tanzania. In this analysis, we considered acceptance of the HIV test by clients to be a measure of acceptability, and number and proportion of women being offered the HIV test to be a measure of effectiveness. Key questions examined included: did CaCx screening clients accept HIV testing when offered? Did the integration of HIV testing result in significantly expanded uptake of HIV testing among eligible women? Did the integration result in identifying previously undiagnosed cases of HIV? How consistently has the HIV testing service been offered as part of CaCx screening in these facilities from 2010 to 2013?

## Methods

### Description of the integration model

The Cervical Cancer Prevention (CECAP) program rolled out services by facility in a staggered fashion, beginning in 2010. Facilities were grouped together for training, and at least three health care providers from each facility were trained using a nationally-approved 6 day training on VIA screening and cryotherapy. Among the 75 providers trained, all had already been trained in PITC and were providing HIV testing: 14 providers (19%) worked at HIV Care and Treatment Centres (CTCs) and 7 (9%) were working in multiple departments of the facility, including HIV CTC, outpatient department (OPD) and/or RCH. In addition, all providers working in the onsite HIV CTCs at these facilities received a one day orientation to CaCx screening, emphasizing the importance of referral for HIV-infected women.

CaCx screening and HIV testing are always provided on the same visit and in the same location. Most facilities described offer CaCx screening in the RCH clinic at least twice a week, but not every day. Family planning (FP) services are offered through the RCH, and are available every day that CaCx screening is offered, and while the service is not integrated (meaning that FP is not routinely offered to every CaCx screening client) the service is at least conveniently co-located. Following national policy, HIV testing is offered to all women unless they are HIV positive or (self) report being tested in the last three months. In Tanzania, the date and results of an HIV test are recorded anonymously in a register, not in individual client records. Therefore, providers do not have a recorded means to share information about HIV testing of a client, and must rely on client’s self report. If a client is reactive to an HIV test administered at the CaCx screening, she is referred to the onsite HIV CTC. Women referred in from the HIV CTC are instructed to show their CTC enrolment card to the provider at the CaCx screening in the RCH. The provider then proceeds with screening without offering an HIV test and notes some of the client’s information, including HIV status, available CD4 counts and whether or not the woman is on antiretroviral therapy, on the CaCx individual client record.

### Sample

This analysis includes routine client level service delivery data from a convenience sample of 21 government health facilities, all of which offered VIA and same-day cryotherapy at the RCH clinic and also had an onsite HIV CTC. All were supported by a United States Government (USG)-funded program to improve maternal, newborn, and reproductive health care services in Tanzania called Mothers and Infants, Safe Healthy and Alive (MAISHA). The 21 facilities are located in four of Tanzania’s 30 regions (Morogoro, Iringa, and Njombe regions and Dar es Salaam municipality). The facility sample included the national consultant referral hospital, two regional hospitals, twelve district hospitals, and six health centres.

In three facilities in this sample, CaCx screening and treatment service was launched before the MOHSW adopted the integration of PITC into the service. This analysis is restricted to the time period in which these facilities had integrated PITC into the CaCx screening service.

### Data collection

Health care providers record clinical information related to the CaCx service on a nationally-approved individual client record at the time of service. ‘Reason a client was not offered an HIV test’ and ‘reason for declining a test’ are fields on this record. Client-level data is available in a client-level database from MAISHA facilities which is maintained by an implementing partner on behalf of the MOHSW; this CaCx prevention program (CECAP) database holds individual client records, stripped of all identifying information. Client records do not leave the health facility and are directly entered by a program-based data manager on site. An aggregated database is housed at the MOHSW to monitor national results on CaCx screening services, however, the aggregated database does not capture responses to the above variables.

Results presented in this paper are drawn from new client records in the CECAP database from August 2010 to September 2013. All new client records within these dates were included in the analysis (no records were excluded). Returning clients were not included in this analysis and will be examined in a separate analysis. Mean months services of service were drawn from the database and cross-checked with program reports. Information on health care providers, such as dates trained, demographic information, and cadre were drawn from program training records.

### Data analysis

Frequencies were run on client level data exported from the CECAP database into Excel and SPSS (version 22). We calculated the percentage of eligible clients who were offered an HIV test, the percentage of women who accepted that offer, and the percentage of women who tested positive for HIV. We also analysed the reasons why eligible clients were not offered or refused an HIV test.

### Ethical considerations

A non-research determination was obtained from John Hopkins University Institutional Review Board (IRB) for secondary analysis of the de-identified CECAP client dataset. The analysis was performed under the guidance of the MOHSW.

## Results

### Characteristics of CECAP providers and clients

A total of 75 providers were trained to offer VIA screening and cryotherapy at the 21 facilities described, from Morogoro, Njombe, Iringa regions and Muhimbili National Referral Hospital in Dar es Salaam. The majority were nurses (71%), and the remainder were physicians (21%), assistant medical officers (7%) and medical attendants (1%). In 18 of the 21 facilities, at least one of the providers trained was stationed at the HIV CTC, or was the medical officer in charge of the HIV CTC. All of the other providers trained were stationed in the RCH clinic (Table 1).

**Table 1:** **Health care providers trained in VIA and cryotherapy and their station in the facility**

Region	Number of providers trained in VIA and cryotherapy from:			Total
	Reproductive and Child Health Clinic (RCH)	HIV Care and Treatment Centre (CTC)	Outpatient Department (OPD)	
Morogoro	22	4	3	29
Iringa	15	3	1	19
Njombe	11	4	1	16
Dar es Salaam (Muhimbili)	8	3	0	11
Total	56	14	5	75

On average, 21 months of service delivery records are included for each facility, and the months of data analysed ranged from 4 to 38 (Table 2). A total of 24,966 women were screened for cervical cancer for the first time at these 21 facilities. The mean age of the women screened was 37.6 years (range 17–88), and mean parity was 3.5 (range 0–16).

**Table 2:** **Health facilities and clients included in the analysis, by region, August 2010 – September 2013**

Region	Number of health facilities	Mean number of months CaCx screening and treatment services were offered (range)	Women screened for cervical cancer		
			Total number	Percent distribution by HIV status*	
				Positive	Unknown
Morogoro	9	29 (6– 38)	12,292	18%	82%
Iringa	6	17 (5 – 30)	5,661	35%	65%
Njombe	5	13 (6 – 19)	5,369	36%	64%
Dar es Salaam	1	4	1,644	21%	79%
Total	21	21 (4 – 38)	24,966	26%	74%

**Figure 1:**
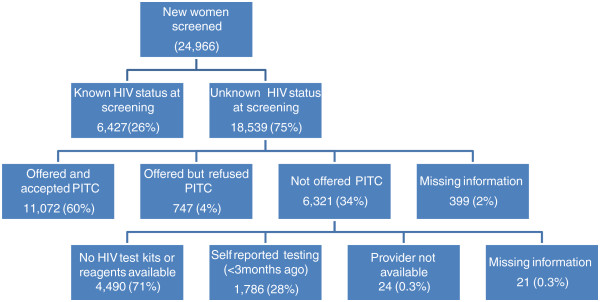
**Coverage and results of HIV testing among CaCx screening clients in Tanzania, August 2010 – September 2013.**

*HIV-positive includes women who presented a CTC enrolment card. All other women were regarded as unknown HIV status (figures on those women testing positive are presented in Figure 1).

The total number of women screened for CaCx increased each quarter as additional facilities introduced the screening service. Peaks occurred around training events, when women were actively recruited to ensure that providers had sufficient practice. The number of women screened monthly at each facility ranged from 25 to 411.

### HIV status and testing

Of the 24,966 clients screened for cervical cancer, 26% (6,427) were referred from the HIV CTC and were not offered an HIV test when they came for CaCx screening. The proportion of clients referred from the HIV CTC varied considerably between regions, ranging from 18% to 36% (Table 1). The remaining 18,539 clients were of unknown HIV status. Of these, 60% were offered an HIV test and accepted. Providers did not offer an HIV test to 4,490 women due to stock outs of HIV test kits or reagent. Another 1,786 women were not offered an HIV test because they self-reported an HIV test within the previous three months (Figure 1).

Almost all (94%) of the 11,819 clients who were offered an HIV test accepted it. Five percent of those testing (582 women) were diagnosed as being HIV infected. Of the 747 women who declined the offer of an HIV test, most (96%) said they ‘did not feel ready’ or ‘were afraid.’ Three percent said they wanted to consult their husband, while the remaining 1% did not specify a reason.

**Figure 2:**
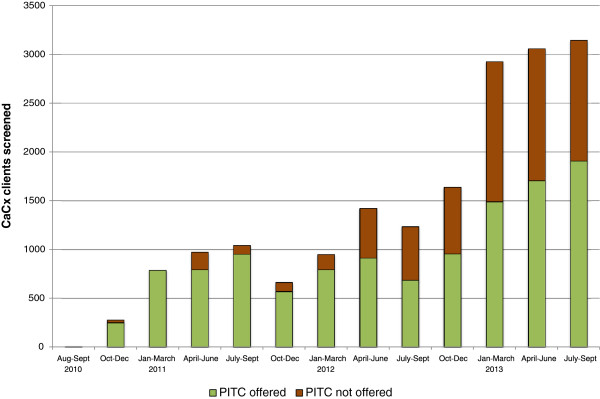
**CaCx screening clients with unknown HIV status who were and were not offered PITC, August 2010 – September 2013.**

The proportion of CaCx screening clients offered an HIV test each quarter varied widely over time, ranging from 16% to 96%. The proportion of clients offered HIV testing started out high, averaging 86% across all facilities in 2011, and fell to 62% in 2012 and 55% in 2013 (Figure 2). From January 2012 onwards, the effects of major, national scale shortages of HIV test kits became apparent in CaCx services. For example, in April 2012, the number of CaCx screening clients *not* offered an HIV test almost quadrupled.

## Discussion

This analysis shows that the model used in Tanzania for integrating HIV testing into cervical cancer prevention services holds great promise. The approach was widely accepted by both providers and clients; providers consistently offered HIV tests when test kits were available; and women almost universally agreed to HIV testing when offered. This finding is consistent with other studies that show high rates of acceptance of HIV testing as part of integrated services [23, 31, 32]. The program reached a large number of women, identified cases of HIV among women who would not otherwise have learned their status, and linked these women with HIV care and treatment.

The biggest challenge affecting the effectiveness of integration was insufficient supply of HIV test kits at the health facilities providing the service. Scaling up HIV testing as part CaCx screening will require additional forecasting, ordering, distribution, and quality monitoring of test kits [23]. In Tanzania, the government-run Medical Stores Department (MSD) manages procurement and logistics of HIV test kits for public health facilities, and notable challenges in supply chain management have been described.

Beginning in December 2011, there was a major disruption of the supply chain for HIV test kits in Tanzania after the first-line HIV test was discontinued due to quality control issues. Throughout 2012 and early 2013, the supply of HIV test kits remained erratic while the MOHSW established a new algorithm, secured the necessary test kits, retrained providers, and began shipping new kits to facilities through regional medical stores [33]. Service delivery data show that this disruption caused a precipitous drop in PITC. According to anecdotal reports, the impact varied at individual facilities, depending on how quickly their stocks of test kits ran out; as supplies of test kits dwindled, health care providers often prioritized pregnant women to facilitate prevention of mother-to-child transmission of HIV.

Although not covered in this analysis, observations from program implementers suggest that a shortage of health care providers in these facilities contributed to both lower than expected number of women screened for CaCx, as well as lower coverage of HIV testing within the service. Like other countries in sub-Saharan Africa, Tanzania suffers from a severe shortage of health care providers; only 40% of the health care professionals needed for adequate coverage of health services are actually employed [34]. The rapid scale up of HIV/AIDS testing, care, and treatment has increased demand for providers, led to heavier workloads, eroded morale, and contributed to burnout [35]. According to anecdotal reports, providers consistently complained about overwork (although they did not specifically point to PITC as the cause). While some HIV-related services in the Tanzanian health system are associated with financial incentives, such as overtime pay, CaCx screening services are not. Declining morale among providers, lack of financial incentives, as well as turnover and reassignments of health care providers may partially explain the low numbers of women screened for CaCx in Tanzania. None of these challenges are unique to Tanzania. In Zimbabwe, a recent study blamed shortages of trained staff, counselling space, and HIV test kits for hampering implementation of PITC [24].

If integrated CaCx and HIV services are to be scaled up nationwide, the CECAP program in Tanzania must ensure that facilities are accurately forecasting demand and ordering sufficient HIV test kits to assure supplies at facilities. The national procurement and commodity logistics systems of MSD for HIV test kits must be improved. Additionally, as PITC is brought to scale, women tested for HIV throughout the health facility—not just those enrolled at HIV CTCs—should be referred for CaCx screening. The program must also find a way to address gaps in the workforce, whether through training, task shifting, and/or efforts to improve retention [10].

Ideally, the integration of CaCx and HIV services should flow in two directions. On the one hand, women coming for CaCx screening should be offered HIV tests and referrals for care and treatment if found positive, and case management of clients with CaCx or pre-cancer should take into consideration the women’s HIV status. On the other hand, all women enrolled in HIV care and treatment should be accessing CaCx screening services, due to their elevated risk of CaCx and need for screening at a younger age. This analysis only examined the first type of integration: that of HIV testing into the CaCx service. Program data suggest, however, that integration in the other direction was not as effective: in these same facilities, the proportion of women enrolled in HIV CTCs who were screened for CaCx ranged from almost none (3%) to less than half (46%) during the April 2010 to March 2012 time period [36]. For a more comprehensive assessment of HIV/ CECAP service integration, further investigation is needed into CaCx screening coverage among women enrolled in HIV CTCs.

### Strengths and limitations

This secondary analysis demonstrates the potential of working with routinely collected service delivery data, especially when steps have been taken to strengthen the health information management system. In this case, it allowed us to assess measures of feasibility and effectiveness of integrating HIV testing into CaCx services in the absence of a formal evaluation. Although analysis was conducted by MAISHA program, which led the program, the calculations presented in this paper were done by individuals who had limited connection to the clinical service. While the paper-based data collection system at the facility level is subject to the normal errors inherent in a manual system in an understaffed health care setting, the MAISHA program led quality improvement measures focusing on providers’ recording of information into client records. This means that the data taken from the individual client records was of somewhat higher quality than typical manually recorded health information data. Data on clients’ reasons for declining an HIV test may have additional constraints because it was solicited and recorded by providers, which may have introduced bias into responses.

## Conclusions

The MOHSW’s CECAP program in Tanzania has piloted systematic integration of HIV testing in CaCx prevention and treatment. The approach has proven to be highly acceptable, both to providers and to women, and an effective way to identify women who do not know they are HIV-infected and refer them to appropriate care and treatment. Countries with a high HIV prevalence, like Tanzania, should prioritize the integration of PITC into CaCx prevention services. Failing to do so will result in a less comprehensive CECAP service, higher costs to clients in terms of illness and mortality, and potentially, higher costs to the health care system for treatment rather than prevention. Successfully implementing this strategy requires recognizing and overcoming commodity supply challenges.

## Abbreviations

CaCx: Cervical Cancer
CECAP: Cervical cancer prevention
CTC: Care and treatment centers
FP: Family planning
HPV: Human Papilloma Virus
LEEP: Loop electrosurgical excision procedure
MAISHA: Mothers and Infants, Safe Healthy and Alive
MOHSW: Ministry of Health and Social Welfare
MSD: Medical Stores Department
OPD: Outpatient department
PITC: Provider initiated testing and counselling
RCH: Reproductive and child health
USG: United States Government
VIA: Visual inspection with acetic acid.
